# Levosimendan may improve survival in patients requiring mechanical assist devices for post-cardiotomy heart failure

**DOI:** 10.1186/cc3979

**Published:** 2006-01-13

**Authors:** Jan-Peter Braun, Dominik Jasulaitis, Maryam Moshirzadeh, Ulrich R Doepfmer, Marc Kastrup, Christian von Heymann, Pascal M Dohmen, Wolfgang Konertz, Claudia Spies

**Affiliations:** 1 Consultant, Department of Anesthesiology and Intensive Care, Campus Charité Mitte, Charité University Hospital, Charité – University Medicine Berlin, Germany; 2 Medical Doctor, Department of Anesthesiology and Intensive Care, Campus Charité Mitte, Charité University Hospital, Charité – University Medicine Berlin, Germany; 3 Consultant, Department of Cardiac Surgery, Campus Charité Mitte, Charité University Hospital, Charité – University Medicine Berlin, Germany; 4 Professor of Cardiovascular Surgery, Director of the Department, Department of Cardiac Surgery, Campus Charité Mitte, Charité University Hospital, Charité – University Medicine Berlin, Germany; 5 Professor of Anesthesiology, Director of the Department, Department of Anesthesiology and Intensive Care, Campus Charité Mitte, Charité University Hospital, Charité – University Medicine Berlin, Germany

## Abstract

**Introduction:**

Most case series suggest that less than half of the patients receiving a mechanical cardiac assist device as a bridge to recovery due to severe post-cardiotomy heart failure survive to hospital discharge. Levosimendan is the only inotropic substance known to improve medium term survival in patients suffering from severe heart failure.

**Methods:**

This retrospective analysis covers our single centre experience. Between July 2000 and December 2004, 41 consecutive patients were treated for this complication. Of these, 38 patients are included in this retrospective analysis as 3 patients died in the operating room. Levosimendan was added to the treatment protocol for the last nine patients.

**Results:**

Of 29 patients treated without levosimendan, 20 could be weaned off the device, 9 survived to intensive care unit discharge, 7 left hospital alive and 3 survived 180 days. All 9 patients treated with levosimendan could be weaned, 8 were discharged alive from ICU and hospital, and 7 lived 180 days after surgery (*p *< 0.002 for 180 day survival). Plasma lactate after explantation of the device was significantly lower (*p *= 0.002), as were epinephrine doses. Time spent on renal replacement therapy was significantly shorter (*p *= 0.023).

**Conclusion:**

Levosimendan seems to improve medium term survival in patients failing to wean off cardiopulmonary bypass and requiring cardiac assist devices as a bridge to recovery. This retrospective analysis justifies prospective randomised investigations of levosimendan in this group of patients.

## Introduction

Failure to wean off cardiopulmonary bypass (CPB) secondary to severe post-cardiotomy cardiac failure is a serious problem in cardiac surgery. Adherence to clearly defined treatment standards has been shown to be advantageous [1-3]. Phosphodiesterase inhibitors and catecholamines are routinely used to treat this complication [4]. In addition to pharmacotherapy, circulation is supported by intraaortic balloon pumping (IABP) and a trial of delayed sternal closure can be indicated [5]. In ongoing severe cardiac failure, mechanical assist devices are used [1-3]. Aims of therapy with these devices are maintenance of adequate perfusion of vital organs and a temporary reduction of cardiac work [6]; centrifugal pumps are commonly used for this purpose. From 49% to 71% of patients are successfully weaned off these devices, and hospital survival is quoted as ranging from 22% to 52% [7-16]. The most commonly encountered complications are bleeding, thrombosis, emboli, renal failure and ongoing severe cardiac failure. Little is known about long term survival of patients treated with assist devices for post-cardiotomy heart failure [10].

Our institution, which treats, on average, 1,500 adult patients per year undergoing CPB, introduced levosimendan for treatment of this complication in February 2003. Compared with other inotropes, levosimendan increases myocardial oxygen consumption to a much lesser extent. There seems to be a particularly marked action on stunned myocardium [17]. The intracellular concentration of calcium is not influenced by levosimendan. The second effect of levosimendan is general vasodilation, which leads to an increase of coronary and splanchnic blood flow [18,19]. Levosimendan induced vasodilation is achieved through opening of the ATP-dependent potassium channels of smooth muscle cells, which is one of the most important factors in protecting tissue from ischemia/reperfusion injury [20]. This mechanism is also responsible for the anti-ischemic, cardio-protective properties of levosimendan [21]. Due to active metabolites, the pharmacological effects of levosimendan are present for many days.

We conducted a retrospective review of the outcome of consecutive patients requiring mechanical ventricular assistance with a centrifugal pump for post-cardiotomy heart failure before and after we started using levosimendan for treatment of severe post-cardiotomy heart failure.

## Materials and methods

We reviewed the charts and data derived from an electronic patient data management system (COPRA™, Leipzig, Germany) of 41 consecutive adult patients undergoing implantation of a centrifugal assist device as a bridge to recovery. Three patients died due to vasoplegia resistant to treatment within the operating room (two conventional treated patients and one patient in the levosimendan group); these patients were excluded from further analysis. From 1 July 2000 to 31 December 2004, 38 patients were included in this analysis and were observed up to 1 June 2005. We determined details of the treatment during the intensive care unit (ICU) stay; if patients were discharged from hospital, we contacted the patients and if necessary their general practitioners by phone to establish survival status.

Our bypass and anesthesia management has been described in our standard operating procedures [22]. In short, for induction of anesthesia we used etomidate, midazolam, fentanyl and pancuronium, and for maintenance of anesthesia fentanyl infusion and isoflurane or sevoflurane were used. CPB was performed using a centrifugal pump (Rotaflow™, Jostra, Hirlingen, Germany) with a pump flow above 2.6 l·min^-1^·m^-2 ^and arterial line pressure above 60 mmHg in normothermia. Pump prime consists of balanced electrolyte solution and hydroxyethyl starch. All patients receive 50,000 KIU·kg^-1 ^aprotinin at the start of CPB. Cardioplegia is induced and maintained with intermittent antegrade warm potassium enriched blood [23]. During CPB we maintain the hematocrit above 22% by packed red blood cell transfusions.

**Figure 1:**
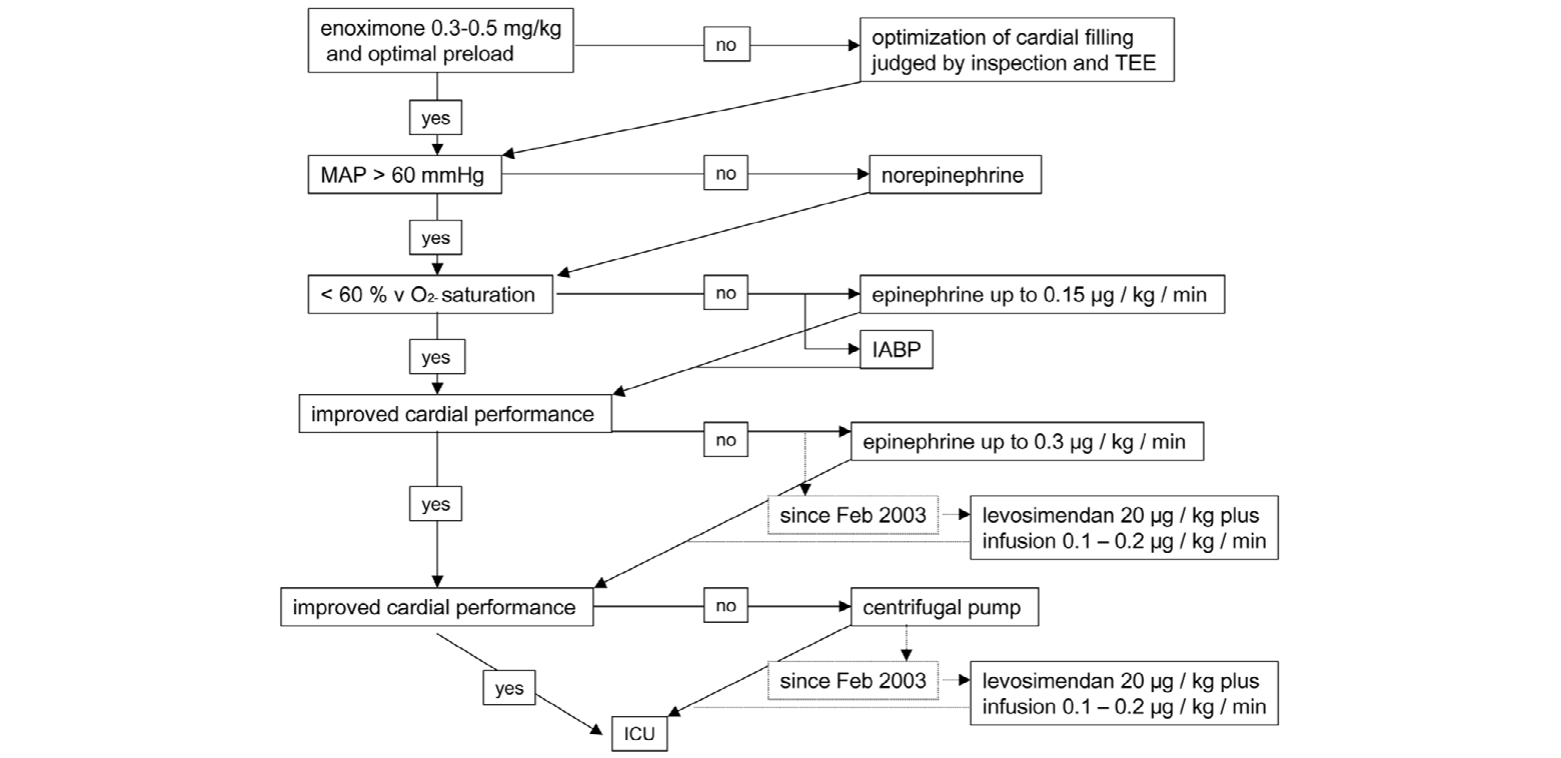
Treatment protocol of the weaning off from cardiopulmonary bypass in patients with acute or chronic impaired cardiac function. IABP, intra-aortic balloon (contra-)pulsation; ICU, intensive care unit; MAP, mean arterial pressure; TEE, trans-esophageal echocardiography; v O_2 _saturation, venous oxygen-saturation.

During termination of CPB all patients described were monitored using transesophageal echocardiography (TEE) using a Sonos 4500 or 5500 System (Hewlett Packard, Andover, Massachusetts, USA) and a pulmonary artery catheter with continuous oxymetry measurement device (Opticath, Abbott, North-Chicago, Illinois, USA). During this period we infused vasoactive substances in every patient according to institutional standards (Figure 1): norepinephrine was applied to maintain mean arterial pressure above 60 mmHg; and in patients with seriously impaired pump function, enoximone 0.3 to 0.5 mg·kg^-1 ^was applied as a slow bolus injection during cardiac reperfusion. If further inotropic support was needed (mixed venous oxygen saturation below 60% and/or cardiac index below 2.4 l·min^-1^·m^-2^), the patients received epinephrine to a maximum dose of 0.15 μg·kg^-1^·min^-1^. If it was impossible to discontinue CPB with this regime we implanted an IABP. During a second attempt to discontinue the pump run we increased the epinephrine dose, if required, up to 0.3 μg·kg^-1^·min^-1^. If this attempt was unsuccessful, we proceed to implantation of a centrifugal pump (Medtronic Biomedicus, Minneapolis, Minnesota, USA). This pump bypassed the ventricle with the worst pump function as judged by TEE and invasive hemodynamic monitoring. If necessary, both ventricles were supported. Goals of hemodynamic therapy were a mixed venous oxygen saturation of 70% or more, and a mean arterial pressure of 70 mmHg. Furthermore, pump flow and filling pressures were adjusted in such a manner that slight opening movements of the aortic valve were visible (during TEE) to avoid intra-cardiac thrombus formation. Epinephrine infusion was guided by the status of the supported ventricle; if possible, doses of less than 0.1 μg·kg^-1^·min^-1 ^were used. First line inotropic support was with enoximone 3 to 5 μg·kg^-1^·min^-1^, reduced to 1 μg·kg^-1^·min^-1 ^in renal failure. Heart rate was limited to 100 beats per minute using esmolol and metoprolol. In new onset atrial fibrillation, electric cardioversion was attempted. IABPs were triggered by the ECG and 1:1 support was chosen. Blood component therapy was guided by coagulation analysis. Heparin was used to maintain activated partial thromboplastin time (aPTT) around 50 seconds once postoperative blood loss had diminished to less than 50 ml·h^-1^. The access wound was closed using a plastic membrane or skin sutures depending on the individual situation.

In February 2003, we added levosimendan to our therapy regime as a last pharmacological option. We started applying the drug in five patients in the operating room during the attempts to wean off CPB and, in four patients, levosimendan therapy was started in the ICU, that is, once the assist had been inserted. We applied an initial bolus dose of 20 μg·kg^-1 ^over ten minutes and then continued therapy with 0.1 to 0.2 μg·kg^-1^·min^-1 ^for a maximum of 48 hours because of the accumulation of active metabolites [24]. Administered in this dosage it has been shown that the pharmacological effects of levosimendan's active metabolites last for at least one week after the end of application. This dosage was chosen due to the advantageous experiences observed in the LIDO-study [25].

The indication for explantation of the assist system was a successful trial of clamping the device for five to ten minutes without deterioration of the hemodynamic parameters. A mixed venous saturation of 70% or more and a mean arterial pressure of 65 mmHg or more had to be maintained using a dosage of epinephrine of 0.1 μg·kg^-1^·min^-1 ^or less. In this clinical situation every patient received a continuous infusion of enoximone.

Statistical evaluation of our retrospective data was done using SPSS version 12.0 (IAC, Chicago, Illinois, USA). The Mann-Whitney *U *test was used to make inter group comparisons. To calculate significance in survival differences between groups chi-square-tests were performed. Significance was assessed at *p *≤ 0.05.

## Results

Demographic data and coexisting diagnoses are given in Table 1. Surgical characteristics are given in Table 2. Survival in relation to treatment with levosimendan is shown in Table 3.

**Figure 2:**
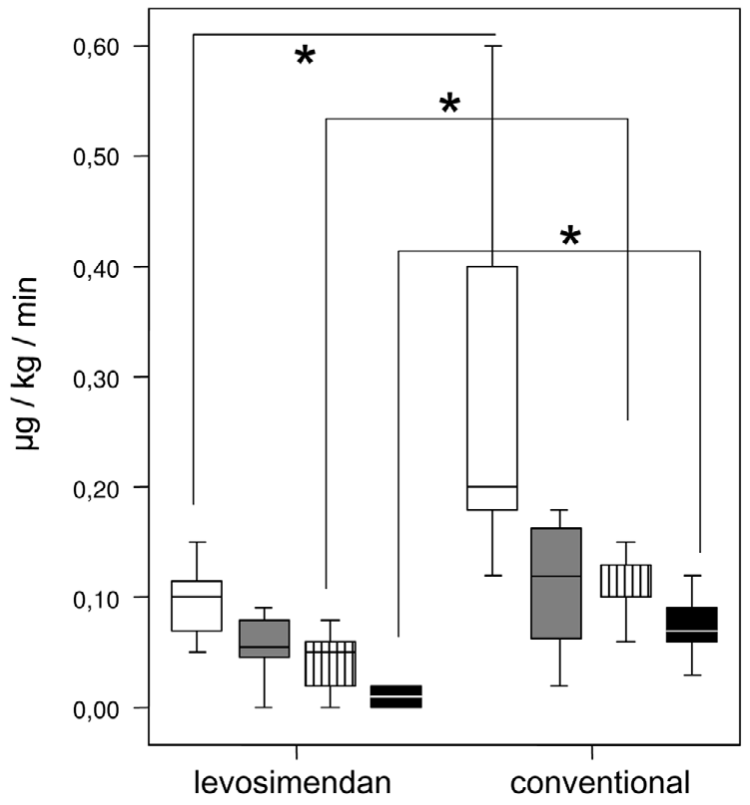
Epinephrine dosages of the intensive care unit survivors. Boxplots presents medians and 25 and 75 percentiles; limits are the 95 confidence intervals. White boxplots are the values of the maximal epinephrine dosage at the day of surgery, grey boxplots are the mean epinephrine dosages during assist device, striped boxplots are the maximal epinephrine dosage during the first 24 hours after explanation of the assist device and black boxplots are the values of the mean epinephrine dosage during the first 24 hours after explantation of the assist device. Asterisks indicate *p *< 0.001 between the groups.

There was no relevant difference between the two groups with regard to treatment with enoximone. The average norepinephrine dosages during infusion of levosimendan were not significantly different between patients treated with or without levosimendan: median 0.14 μg·kg^-1^·min^-1 ^(interquartile range 0.18) in the levosimendan group; and median 0.2 (interquartile range 0.28) in the conventional treatment group. On the day of primary surgery, the maximum dose of epinephrine was higher in patients not receiving levosimendan. After explantation, levosimendan patients required less epinephrine (Figure 2). The median of the norepinephrine dosage after explantation was 0.06 μg·kg^-1^·min^-1 ^(interquartile range 0.012) in the levosimendan treated patients versus 0.14 μg·kg^-1^·min^-1 ^(interquartile range 0.4) in the conventional treated patients (*p *= 0.03).

**Figure 3:**
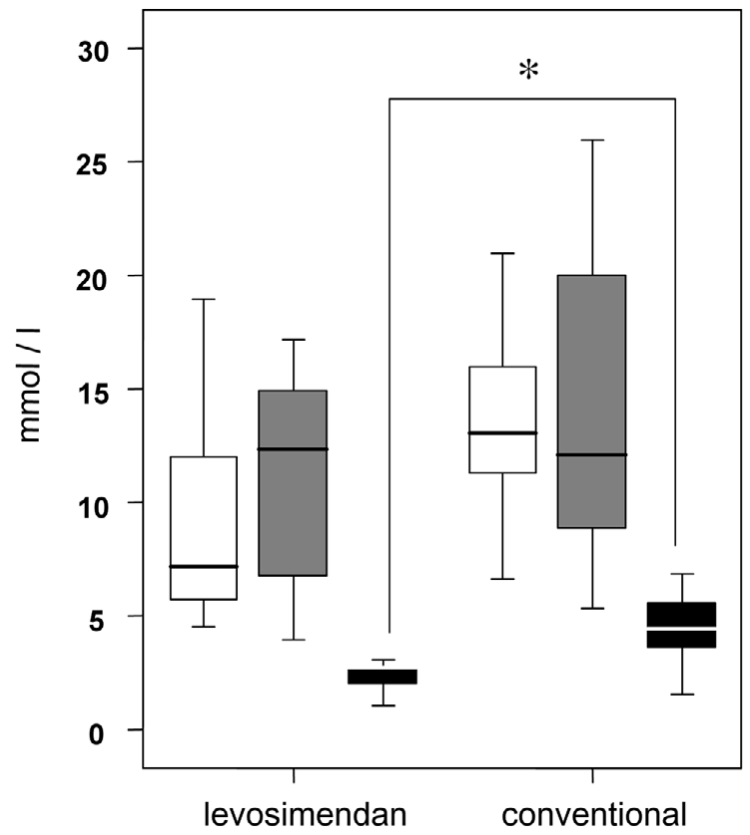
Plasma lactate concentrations. Boxplots presents medians and 25 and 75 percentiles; limits are the 95 confidence intervals. White boxplots are the values of the maximal lactate concentrations during surgery, grey boxplots are the values of the maximal lactate concentrations during assist device, and black boxplots are values of the maximal lactate concentrations after discharge from assist device. Asterisks indicate *p *= 0.016 between the groups.

After explantation of the assist device, plasma lactate concentrations were significantly lower in the levosimendan group (Figure 3).

**Figure 4:**
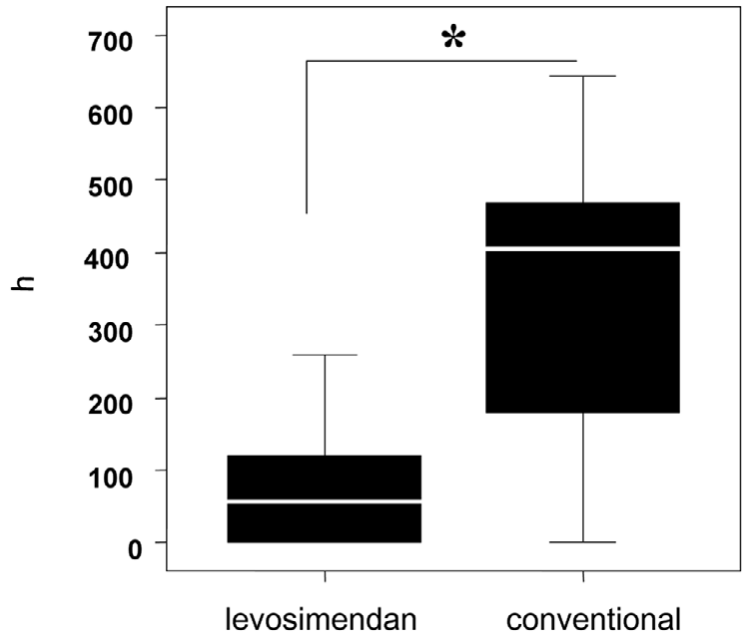
Duration of the extracorporal renal replacement. Boxplots present medians and 25 and 75 percentiles; limits are the 95 confidence intervals. The asterisk indicates *p *= 0.023 between the groups.

There was no significant difference in duration of mechanical cardiac assistance with either centrifugal pumps (mean 3.4 days, range 1 to 7 days) or IABPs (mean 6.2 days, range 6 to 53 days). Mean time on mechanical ventilation was 6.2 days (range 2 to 44 days), mean ICU stay was 27 days (range 6 to 53 days), and mean hospital stay was 60 days (range 6 to 196) without any significant difference between the groups. The incidence of renal replacement therapy in both groups was 80%, but there was a large difference in duration of renal replacement therapy between the ICU survivors of the two groups (Figure 4).

## Discussion

In this retrospective observation, we were able to demonstrate a positive effect of levosimendan on 180-day survival rates in patients with severe post-cardiotomy heart failure. The group of patients treated with levosimendan required less epinephrine in the postoperative period, showed lower plasma lactate concentration after explantation of the assist device, and significantly shorter duration of renal replacement therapy. No difference was found between the groups regarding the incidence of acute renal failure as well as ICU or hospital length of stay.

The survival rates in the conventional treatment group were similar to those reported by other centres; 10 studies observing the therapy with assist device implantation as a bridge to recovery showed that weaning was possible in 49% to 71% and discharge from hospital in 22% to 52% of patients [7-16]. In our study, 69% of the patients in the conventional therapy group were able to be weaned from the assist device and 24% were finally discharged from hospital. In contrast, all of the patients in the levosimendan group were successfully weaned and 89% could be discharged from hospital. We have taken into consideration that the patients in the levosimendan group were of significantly younger age than those in the conventional treatment group (Table 1). The average age of the patients in the levosimendan group was 57 years and ranged between a minimum of 45 years and a maximum of 68 years. This corresponds with an average age of 59 years published in other studies.

Not many data exist on long-term survival rates of patients receiving an assist device secondary to severe post-cardiotomy cardiac shock. Hoy and colleagues [10] have reported 62 cases with implanted centrifugal pumps. In that study, 27 of the observed patients were able to be discharged from hospital, 9 died in the first year after discharge, 10 further survived for less than 5 years, 7 survived for 6 to 10 years and 1 patient survived for more than 10 years after the procedure.

The advantageous effects of levosimendan in patients with acute decompensated cardiac failure have been demonstrated in clinical trials. The multicenter RUSSLAN study [25] investigated levosimendan in three different dosages versus placebo in patients with acutely impaired cardiac function due to myocardial infarction. The higher dosage of levosimendan (bolus 24 μg·kg^-1 ^followed by infusion of 0.4 μg·kg^-1^·min^-1^) was associated with increased incidences of hypotension and ischemia compared to patients who received a bolus of 24 μg kg^-1 ^followed by infusion of 0.2 μg × kg^-1 ^× min^-1 ^or patients who received a bolus of 12 μg kg^-1 ^followed by infusion of 0.2 μg × kg^-1 ^× min^-1 ^or patients who received a bolus of 6 μg kg^-1 ^followed by infusion of 0.1 μg × kg^-1 ^× min^-1^. The total number of levosimendan treated patients showed a significantly reduced 14-day mortality (*p *= 0.031) and the 180-day mortality tended to be lower in the levosimendan groups (*p *= 0.053). The multicenter LIDO study [26] investigated levosimendan versus placebo in patients with acute decompensated heart failure; 180-day survival was significantly improved in the levosimendan group (*p *= 0.029). No controlled studies have ever examined the effect of levosimendan administration in cardiac surgical patients with severe impairment of ventricular function. Lehmann and co-workers [27] reported 10 patients with severe cardiogenic shock undergoing emergency coronary artery bypass grafting (CABG). These patients were treated with levosimendan. Two patients died and eight patients showed an excellent clinical outcome without further complications. One study showed a significant improvement of hemodynamic measurements in patients undergoing CABG surgery with normal ventricular function [18].

Morelli and co-workers showed that levosimendan improved the left ventricular stroke work index in septic patients with reduced ventricular function compared to patients treated with dobutamine [19]. The authors observed no difference in norepinephrine dosages between the groups. Their patients were treated with optimal fluid loading. In our retrospective analysis, we also found no increase in administered norepinephrine dosages in levosimendan treated patients. In Morelli's study, levosimendan led to an increase in gastric mucosal blood flow, creatinine clearance, and urinary output. The authors found decreased troponin and lactate concentrations in levosimendan treated patients. In our retrospective observation, the duration of renal replacement therapy was significantly lower in the levosimendan group compared to the conventional therapy group. This could be due to improved renal perfusion.

The significantly lower plasma lactate concentration following device explantation in levosimendan treated patients could possibly be an indicator for improved splanchnic perfusion and for protection of organs from ischemia/reperfusion injury through opening of ATP-dependent potassium channels by levosimendan. Another explanation for this finding could be a reduced administration of epinephrine in the levosimendan group. Epinephrine itself may lead to impaired splanchnic perfusion and metabolic alterations resulting in hyperlactatemia [28].

## Conclusion

The 180-day survival rate in patients receiving an assist device due to severe post-cardiotomy cardiac failure was significantly improved by treatment with levosimendan. A significant reduction of the epinephrine dose was possible. The incidence of hyperlactatemia was lower and the duration of renal replacement therapy was shorter.

The results presented here must be interpreted cautiously since this observation is retrospective. Our data justify future prospective randomised trials of levosimendan in cardiac surgical patients suffering from severe heart failure.

## Key messages

• 	Failure to wean off cardiopulmonary bypass (CPB) secondary to severe postcardiotomy cardiac failure is a serious problem in cardiac surgery.

• 	Adherence to clearly defined treatment standards has been shown to be advantageous.

• 	In this case series the 180-day survival rate in patients failing to wean off cardiopulmonary bypass and requiring cardiac assist devices as a bridge to recovery was about 10% (3 of 29).

• 	The 180-day survival rate in 9 patient who were additionally treated with levosimendan was about 78 % (7 of 9).

• 	Our data justify future prospective randomised trials of levosimendan in cardiac surgical patients suffering from severe heart failure.

## Abbreviations

CABG = coronary artery bypass grafting; CPB = cardiopulmonary bypass; IABP = intraaortic balloon pumping; ICU = intensive care unit; TEE = transesophageal echocardiography.

## Competing interests

The authors declare that they have no competing interests.

## Figures and Tables

**Table 1:** Preoperative characteristics of the patients

Characteristic	Conventional	Levosimendan	*P *value
n	29	9	
Age (years)	68 ± 7^a^	57 ± 8^a^	0.001
Body mass index	27.1 ± 3^a^	26.3 ± 3^a^	0.41
Male:female	19:10	8:1	0.31
LVEF	34 ± 14^a^	33 ± 17^a^	0.96
Coronary artery disease	26	7	0.55
Arterial hypertension	23	7	0.94
Pulmonary arterial hypertension	3	2	0.61
Diabetes mellitus	12	3	0.73
Chronic renal insufficiency	10	2	0.59
Peripheral vascular disease	7	2	0.94
Atrial fibrillation	12	3	0.74
Hyperlipidemia	15	4	0.42
Nicotine abuse	6	4	0.13
COPD	5	2	0.71

^a^Mean ± standard deviation. Mann-Whitney-*U* test was used to determine *p* values between the groups. Chi square test was used to compare categorial data. COPD, chronic obstructive pulmonary disease; LVEF, left ventricular ejection fraction.

**Table 2:** Surgical characteristics of the patients

Characteristic	Conventional	Levosimendan	*P *value
CABG	17	6	0.84
AVR	2	1	0.79
MVR	4	1	0.71
CABG + VR	6	1	0.36
Duration of CPB (minutes)	170 ± 70^a^	176 ± 101^a^	0.65
Duration of AO (minutes)	69 ± 60^a^	80 ± 81^a^	0.76
LVAD	22	7	0.70
RVAD	5	1	0.95
BiVAD	2	1	0.86
Duration of AD (days)	3.2 ± 2^a^	3.8 ± 1.5^a^	0.20

^a^Mean ± standard deviation. Mann-Whitney *U* test was used to calculate p-values between the groups (duration data). Chi square test was used to compare categorial data. AO, aortic cross clamping; AVR, aortic valve replacement; BiVAD, biventricular ventricular assist device; CABG, coronary artery bypass grafting; CABG + VR, CABG combination with a valve replacement; CPB, cardiopulmonary bypass; IABP, intra-aortic balloon pump counter-pulsation; LVAD, left ventricular assist device; MVR, mitral valve replacement; RVAD, right ventricular assist device.

**Table 3:** Survival status of the groups

	Conventional	Levosimendan	*P *value
n	29	9	
AD survival	20	9	*p *= 0.082
ICU survival	9	8	*p *= 0.005
Hosp survival	7	8	*p *= 0.001
180-day survival	3	7	*p *< 0.001

Chi square test was used and *p *values between the groups were determined according to exact Fischer's test. 180-day survival, survival of the first 180 days after surgery; AD survival, survival of assist device; Hosp survival, survival of hospital stay; ICU survival, survival of intensive care unit stay.
